# Philologic Reform

**Published:** 1899-11

**Authors:** 


### PHILOLOGIC REFORM.

THE inconsistency between the spelling and pronunciation of a vast number of words in the English language has always constituted a serious obstacle to the acquisition of the language by foreigners. English alone in the hierarchy of civilized tongues occupies this unexplainable and indefensible position. German is absolutely consistent, a quality which adds much to this flexible and sonorous tongue; Italian and the romance languages generally, with the exception of French, show very little disposition toward squeezing in the superfluous letters. The Slavonic languages or dialects are scarcely as yet worthy of position for comparison, but in spite of the apparent redundancy of consonants, they are less open to criticism than English.

But our language is gradually eliminating these defects and is constantly making progress toward simplification and rational spelling.

In America, one no longer sees the "u" in such words as labor, Savior and the like, though it is still retained in England. The grotesque differences in the pronunciation of the termination "ough" are being overcome by appropriate spelling. Although, through, plough, are orthographized into altho', thru, plow. As yet no one has had the temerity to write sluff, coff, troff, or more consistently sluf, cof, trof. The diphthong is fast disappearing in medical literature from such words as edema, diarrhea, hemorrhage (why not the "h" also from the two last? What concern have we with the Greek rho?) The hyphen is also passing out of use in compound words; final e is being dropped from such words as oxid, morphin, etc.

Several medical journals have adopted in part the reform or fonetic system of spelling and are pruning words of useless exuberance of letters. The *Journal of the American Medical Association*, the *Philadelphia Medical Journal*, the *American Journal of Surgery and Gynecology*, the *Medical World* (the modifications of spelling in the last named probably will not be at once apparent to the bulk of its subscribers), are among the best known of the medical publications which have initiated reform measures in orthography.

Reforms of this character are slow to gain headway, but their establishment is apt to be permanent. Good authority is placing them in literature, and it is probable that another decade will relegate to the limbo of obsoletism many of the orthographic peculiarities of to-day.

Even the disgruntled subscriber who wants his journal "stoped," may, in the not remote future, congratulate himself for having been so far ahead of his times, if still behind on subscription dues. W.

